# Are You What You Read? Predicting Implicit Attitudes to Immigration Based on Linguistic Distributional Cues From Newspaper Readership; A Pre-registered Study

**DOI:** 10.3389/fpsyg.2019.00842

**Published:** 2019-05-03

**Authors:** Dermot Lynott, Michael Walsh, Tony McEnery, Louise Connell, Liam Cross, Kerry O’Brien

**Affiliations:** ^1^Department of Psychology, Fylde College, Lancaster University, Bailrigg, United Kingdom; ^2^Institute for Natural Language Processing, University of Stuttgart, Stuttgart, Germany; ^3^Linguistics and English Language, Lancaster University, Bailrigg, United Kingdom; ^4^Department of Psychology, School of Health and Wellbeing, University of Wolverhampton, Wolverhampton, United Kingdom; ^5^School of Social Sciences, Monash University, Caulfield East, VIC, Australia

**Keywords:** IAT, language, implicit attitudes, bias, implicit association test

## Abstract

The implicit association test (IAT) measures bias towards often controversial topics (e.g., race, religion), while newspapers typically take strong positive/negative stances on such issues. In a pre-registered study, we developed and administered an immigration IAT to readers of the Daily Mail (a typically anti-immigration publication) and the Guardian (a typically pro-immigration publication) newspapers. IAT materials were constructed based on co-occurrence frequencies from each newspapers’ website for immigration-related terms (migrant/immigrant) and positive/negative attributes (skilled/unskilled). Target stimuli showed stronger negative associations with immigration concepts in the Daily Mail compared to the Guardian, and stronger positive associations in the Guardian corpus compared to the Daily Mail corpus. Consistent with these linguistic distributional differences, Daily Mail readers exhibited a larger IAT bias, revealing stronger negative associations to immigration concepts compared to Guardian readers. This difference in overall bias was not fully explained by other variables, and raises the possibility that exposure to biased language contributes to biased implicit attitudes.

## Introduction

Janet is less likely to be called for an interview than John. Jamal is less likely to get a job than James. Obese applicant Jill is less likely to be shortlisted than her non-obese colleague Julia. In fact, you are more likely to experience a negative outcome in a range of life scenarios if you are female, or overweight, or have a stereotypical “black” first name. If this has happened to you, you may have been the victim of implicit bias.

Biased attitudes and prejudicial decision making have wide-ranging, negative effects, and even seemingly inconsequential levels of bias can have a dramatic impact on individual and societal-level outcomes. Evidence suggests that people routinely incorporate unconscious, implicit biases into their decision making, leading to negative outcomes for the affected group in employment, education, criminal justice, politics, and healthcare, where biases can be related to gender, race, age, and many other characteristics (e.g., Moss-Racusin et al., 2012; O’Brien et al., 2013; Greenwald et al., 2015). For example, a tiny, 1% pro-male bias in terms of staff hiring and promotion decisions can quickly lead to a 70:30 gender discrepancy at senior staff levels (Martell et al., 1996; Agars, 2004). Measures of anti-fat bias predict how obese individuals are less likely to be shortlisted for jobs (O’Brien et al., 2013), less likely to be employed than someone who is non-obese (Larkin and Pines, 1979), and even if employed, they are likely to be asked to do more menial tasks (Bellizzi and Hasty, 1998) and will be paid less (Pagán and Dávila, 1997). Similarly, job applications with stereotypical black names, are less likely to be shortlisted relative to applications with stereotypical white names (Bertrand and Mullainathan, 2004). For any marginalized group the implicit attitudes of others can potentially have a profound impact on their chances of success.

Language is central in communicating our thoughts, feelings and attitudes, and as such, the language we produce reflects our preferences and biases. Distributional theories of language and cognition (e.g., Landauer and Dumais, 1997; Lynott and Connell, 2010; Connell and Lynott, 2014), combined with recent advances in corpus linguistics and machine learning, may help us uncover the origins of implicit attitudes and how linguistic experience can enhance or attenuate such views. In this paper, we examine how the way language is used might provide some insights to implicit attitudes, and how people’s differing linguistic experiences might be associated with different levels of implicit bias.

### The Role of Language in Encoding Bias

An increasingly popular way of representing meaning in language is to consider words and phrases as part of a multidimensional semantic space (Landauer and Dumais, 1997; Louwerse and Jeuniaux, 2010; Connell and Lynott, 2013). Such models reflect distributional theories of semantic memory that contend that the co-occurrence patterns of words in language capture at least some of the meaning of those words. In Firth’s (1957) words, “you shall know the meaning of a word by the company it keeps.” Distributional language models have been found to capture a broad range of cognitive phenomena relating to language processing, memory, and mental representation more generally (e.g., Landauer and Dumais, 1997; Louwerse and Connell, 2011; Connell and Lynott, 2013), finding consistent evidence that people are sensitive to statistical regularities that occur in language (Samuelson, 2002; Saffran, 2003). However, it is only recently that researchers have applied distributional models of language to the domain of social cognition (Lynott et al., 2012; Bhatia, 2017; Caliskan et al., 2017), with the surprising possibility emerging that language patterns may be related to implicit biases in society.

Recent work has shown that statistical distributional properties of words reflect human biases and prejudicial judgements (Lynott et al., 2012; Caliskan et al., 2017). The way positive and negative terms are distributed in language closely reflects the positive or negative biases people exhibit toward various concepts, as measured for example, by implicit association tests (IAT). The IAT is a computerized task and is by far the most commonly used measure of people’s automatic associations between concepts (e.g., Greenwald et al., 2003). In an IAT, participants must classify stimuli into categories as quickly as possible, where faster responses indicate that there are stronger associations between those concepts (Arkes and Tetlock, 2004). For example, in a Race IAT, which contrasts prototypical black or white personal names, people consistently respond more quickly to black names paired with negative concepts (e.g., “Jamal” and “disgusting”), compared to white names paired with negative concepts (e.g., “Brad” and “disgusting”), and vice versa for pairings with positive concepts, indicating stronger negative associations in the minds of participants toward black names (Greenwald et al., 1998). When a participant completes an IAT, a D score is calculated based on their response times in the task. A higher value for D indicates a stronger (generally negative) association for the concepts in question. IAT D scores with values greater than 0.15 have been considered “slight,” greater than 0.35 as “moderate,” and greater than 0.65 as “strong” (Haider et al., 2011).

Using corpus analysis, we found that black names are much more likely to co-occur with negative, rather than positive attributes, compared to white names. In fact, for 16 different IATs (e.g., related to topics such as race, obesity, drug use, and others), Lynott et al. (2012) found a strong correlation (*r* = 0.79) between implicit biases predicted by distributional properties of the word stimuli from those IATs, and the actual degree of bias indicated by the human behavioral data (the IAT D score). That is, the greater the discrepancy between, for example, black and white names and co-occurrences with positive/negative attributes, the greater the observed human behavioral bias (i.e., the higher the IAT D score).

More recently, Caliskan et al. (2017) extended this logic and applied cutting-edge machine learning techniques to corpora of hundreds of millions of words to create high-dimensional semantic spaces that represent word meaning in the form of vectors. The degree of association between words can then be calculated by measuring the cosine between any pair of word vectors. Using this technique, Caliskan and colleagues again found striking parallels between human biases in behavioral data on the one hand, and biases observed in the linguistic models on the other. For example, the linguistic information accurately reflected people’s preferences for benign contrasts between flowers and insects, as well as the negative bias that whites show toward stereotypical black names, and even how linguistic associations between gender terms and professions (e.g., level of association between female/male and job terms such as technician/teacher/lawyer etc) predict gender distributions in those professions.

Thus, the findings of both Caliskan et al. (2017) and Lynott et al. (2012), indicate that language itself encodes human biases, which can be recovered using appropriate statistical models. Furthermore, these models apply to a broad range of biases regardless of whether they are morally neutral, socially problematic, or just reflecting the actual state of the world. In this paper, we suggest that if people’s sensitivity to linguistic distributional information is associated with the implicit biases they hold, then it makes sense that groups of people who have different linguistic experience could exhibit differing levels of implicit bias. Specifically, we consider whether reading different newspapers on a regular basis might be associated with different levels of bias on the topic of immigration.

Immigration is an ever-present topic in the media, and previous research has found that implicit attitudes to immigration (as measured by the IAT) reflect people’s explicit views and their support for immigration policies, independent of other measures such as political ideology or socio-economic concerns (Pérez, 2010). Mutz and Goldman (2010, p242) suggest that “the way outgroup members are portrayed in the media is widely believed to have consequences for levels of prejudice and stereotyping in the mass public.” While this assertion may be true, there is little direct evidence to support such a claim.

However, some research is suggestive of a link between media consumption and attitude development Arendt (2010), took a content-analytic approach to investigate the effects of news consumption on implicit attitudes to the European Union. It was found that those who read more negative content about the EU developed more negative implicit associations toward the EU. In a 2012 experimental study, Arendt also found that participants exposed to emotionalised news content describing foreigners as criminals subsequently showed short-term increases in associations between foreigners and criminality, although there was no effect for non-emotionalised content. Arendt and Northup (2015) found that long-term exposure to stereotypical news can increase people’s implicit biases (e.g., of foreigners as criminals). However, they include no measure of bias from actual media content, but rely on the assumption that foreigners are over-represented as criminals in the media, combined with self-reports of how much local TV participants watch or how much tabloid news they consume. By contrast, our current corpus-inspired approach provides objective measures of bias from a given media source, and so provides a step forward in our ability to assess the media’s use of language and how it is related to people’s attitudes. To this end, we conducted a pre-registered study which examined implicit (and explicit) attitudes to immigration based on two mainstream newspapers from the United Kingdom (Daily Mail/Guardian).

Historically, The Guardian and the Daily Mail have displayed contrasting attitudes to immigration. For example, a 2016 Daily Mail commentary (“Who will speak for England?,” 2016), makes reference to “a tsunami of migrants” and the wish to “be a self-governing nation, free in this age of mass migration to control our borders.” Such a stance contrasts with an editorial from the Guardian in 2018 (“May’s immigration regime,” 2018) that describes the government’s stated tactic of creating a “hostile environment” for immigrants, and a general approach to immigration that is “unjust, inhumane, and incompetent.” Evidence for these views representing longer-term trends has been observed using corpus-based and discourse-analytic approaches to evaluating news content (e.g., Gabrielatos and Baker, 2008; Gabrielatos et al., 2010). For example, Gabrielatos and Baker (2008) found that over a 10 year period, the Daily Mail had much higher frequencies of collocations highlighting negative aspects of immigration (e.g., phrases such as “illegal” refugee, “illegal asylum seeker,” and “bogus immigrant”), when compared with the Guardian (p 7).

Given these patterns, and building on previous findings (Lynott et al., 2012; Caliskan et al., 2017), we expected that IAT bias scores would be higher for readers of the Daily Mail compared to the Guardian, in keeping with the linguistic co-occurrence patterns observed on those newspapers’ websites.

## Materials and Methods

### Pre-registration and OSF Project Page

The study outline, data collection, and analysis plans were pre-registered on AsPredicted.org, on October 12th, 2016, and deposited on the project’s website on the Open Science Framework. All data and materials are available here: https://osf.io/dw6n5/.

### Participants

Our target sample was *n* = 200, which would provide >90% power to detect a medium effect size of Cohen’s *d* = 0.5, or >80% power to detect an average effect size for social psychology of *d* = 0.43 (e.g., Richard et al., 2003). Using Qualtrics Panels (a managed panel of participants recruited by Qualtrics), 209 participants completed the study. To avoid participants being aware that we were specifically interested in readers of the Guardian/Daily Mail, we used a screener questionnaire at the beginning of the study. For the screener, participants answered a number of irrelevant questions (e.g., how do you commute to work daily? What is your internet browser of choice?), and only those who selected the Guardian or Daily Mail to the question “What newspaper do you prefer to read?” were directed to complete the rest of the study. Other respondents exited the survey at this point.

Because a technical error allowed participants to complete the study without responding to all of the IAT blocks, the first 26 participants were excluded. One additional participant was excluded for not correctly answering a simple trap question, and following the general data processing procedure for the IAT (Greenwald et al., 2003), a further 22 participants were excluded (e.g., long responses, high rate of non-responding) leaving data from 160 participants for our reported analyses (Daily Mail = 76 and The Guardian = 84). Although we did not specifically control for demographics, both participant samples were reasonably closely matched (values for Daily Mail and The Guardian, respectively): gender (47% female and 48% female), age (45.36 and 40.54 years), level of education (14.28 and 15.52 years), proportion born in United Kingdom (89.5 and 83.3%).

### Materials

We created an IAT on the topic of immigration. The Daily Mail is known for its anti-immigration stance, while The Guardian is seen as having more liberal views (e.g., KhosraviNik, 2010). We therefore created a set of IAT stimuli to reflect these observed differences in attitudes between the Daily Mail and Guardian newspapers. To achieve this difference, we first created a list of immigration related terms (e.g., migrant and foreigner) and a set of positive and negative attributes (skilled/unskilled and fantastic/terrible), combining items from previous studies (e.g., Nosek, 2005), with additional items which were close synonyms, but which tended to have higher frequencies of occurrence (e.g., terrible/horrible). We then calculated the co-occurrence frequencies within documents for each combination of the immigration terms and the attribute words (i.e., the number of times each immigration term occurred in a document with an attribute term). Co-occurrences were calculated using Google web search, restricted to the domains of guardian.co.uk and dailymail.co.uk. Document co-occurrence counts were calculated independently by two of the authors in September, 2016, and found to be in agreement (i.e., the same counts were obtained independently). We then created a single set of IAT stimuli where the words had more frequent immigration-negative associations (and less frequent immigration positive associations) in the Daily Mail than the Guardian. The final set of words used were immigration terms (migrant, refugee, outsider, alien, and expatriate), non-immigration terms (native, citizen, local, national, and resident), positive terms (beautiful, happy, fantastic, skilled, and lovely), negative terms (illegal, invasion, terrible, burden, and filth).

While not central to our primary hypotheses, we also collected some supplementary measures, and report additional analyses that consider the relationship between the level of implicit bias and (1) explicit attitudes to immigration, (2) previous voting behaviors (political party voted for in previous General Election; vote to Leave/Remain in EU Brexit referendum), and (3) the death penalty. For (1), in previous work on immigration, Pérez (2010) found that while explicit measures of attitudes were strongly correlated with each other (i.e., attitudes to Latino and White immigrants), there was differentiation in the relationship between explicit and implicit measures – there was a non-significant relationship between explicit and implicit attitudes to white immigrants, while the relationship for Latino immigrants was positive, and significant. More generally, there is often wide variation in the strength of association between explicit and implicit measures on the same topic (see Nosek, 2005), although the relationship does tend to be positive. Regarding (2), voting preferences have often been found to be related to attitudes to immigration (e.g., Blinder and Allen, 2016; Harteveld et al., 2017; Blinder and Richards, 2018). For example, if people switch party allegiance, their attitudes to immigration tend to follow the new party’s views (Harteveld et al., 2017). Immigration was also a key topic during the Brexit campaign to remain in or leave the EU, and motivated many people to vote in a particular direction (e.g., Goodwin and Heath, 2016; Goodwin and Milazzo, 2017). Lastly, on (3), it was previously observed that attitudes to the death penalty were strongly correlated to voting preferences in the Brexit referendum (e.g., Kaufmann, 2016), such that people with stronger attitudes in favor of re-introducing the death penalty were also more likely to vote for the United Kingdom to leave the European Union. We felt that the inclusion of these measures may provide some insights and/or highlight patterns that may give rise to specific predictions to be tested in future work.

### Procedure

Following the screener questionnaire (see above), participants read the participant information page, which gave an overview of the study, and then indicated their informed consent. Participants then completed the Immigration IAT, followed by the explicit questions regarding views on immigration, political party preferences, and past voting behavior. See Supplementary Information for the full list of questions used. Participants indicated how often they read a range of newspapers, and also to what extent they trusted their content. Participants were also asked for some basic demographic information (age, gender, whether they were born in the United Kingdom or not, level of education), and finally they were brought to a written debrief page. Participants were given the option of withdrawing from the study within 2 weeks if they emailed the researcher with an anonymised participant ID.

### Data Analyses

An IAT D score was calculated for each participant, based on the steps described in Greenwald et al. (2003), with higher *D* values indicating a greater level of negative associations with immigration-related concepts. As outlined in our pre-registration document, *D* values were submitted to an analysis of variance, and a follow up ANCOVA controlling for level of education. Although our predictions were directional, we did not explicitly pre-register one-tailed tests, so report two-tailed *p*-values for the confirmatory analyses. As a secondary analysis, we pre-registered that the effect may be larger for those who rely more exclusively on the Guardian or Daily Mail as their main source of news.

Confirmatory analyses (specified in our pre-registration document) are reported first in the results section, while any additional, non pre-registered analyses are reported in a subsequent Exploratory Results subsection. Findings for exploratory analysis should always be treated with caution, as conducting multiple exploratory analyses increases the possibility that “significant” findings are in fact false positives. Nonetheless, observed patterns may provide useful insights or suggestions for future research directions.

## Results

### Confirmatory Results

**Figure 1:**
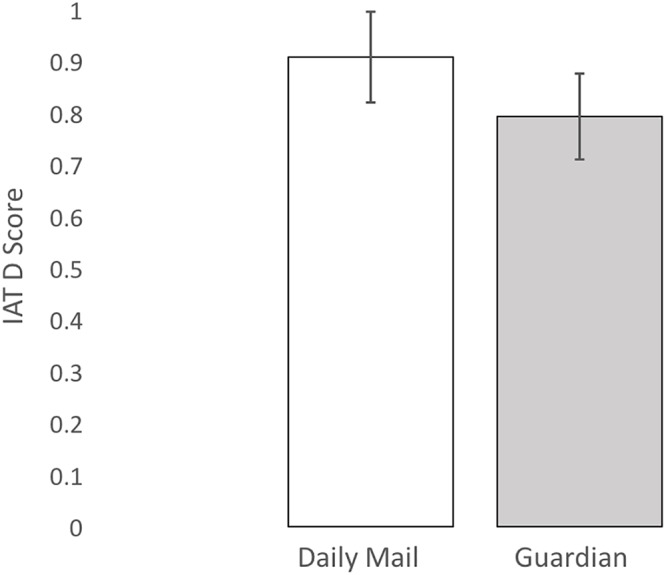
Difference in IAT scores between readers of the Daily Mail and Guardian newspapers. Error bars show 95% confidence intervals. Image from doi: 10.17605/OSF.IO/DW6N5

Overall, Guardian readers (*M* = 0.79, *SD* = 0.42) had lower IAT scores than Daily Mail readers (*M* = 0.91, *SD* = 0.34) – *F*(1,158) = 3.526, MSE = 0.148 *p* = 0.062, and *d* = 0.31 (considered a small-to-moderate effect size: Cohen, 1988. See Figure 1). Running the same analysis with Years of Education as a covariate does not dramatically change the effect of newspaper [*F*(1,157) = 2.923, MSE = 0.149, and *p* = 0.089], and Education itself did not affect IAT scores – *F*(1,157) = 0.13, MSE = 0.149, and *p* = 0.724.

In a follow-up analysis of regular readers, the effect of newspaper is larger: participants who read the Guardian at least once a week (*N* = 69, i.e., including all those who read the paper daily; more than once per week; or once per week) had lower bias scores (*M* = 0.747, *SD* = 0.41) than those who read the Daily Mail at least once a week (*N* = 68, *M* = 0.911, *SD* = 0.35) – *F*(1,135) = 6.299, MSE = 0.146, *p* = 0.013, *d* = 0.43. The effect of Newspaper persists when Years in Education is included as a covariate – *F*(1,134) = 5.938, MSE = 0.147, *p* = 0.016, with Education having no reliable effect (*p* = 0.931). The pattern is similar if we consider only those readers who read one newspaper, but not the other: participants who read the Guardian at least once a week, but never the Daily Mail (*N* = 29) had lower bias scores (*M* = 0.65, *SD* = 0.48) than those who read the Daily Mail at least once a week (*N* = 29), but never the Guardian (*M* = 0.89, *SD* = 0.40) – *F*(1,56) = 4.245, MSE = 0.198, *p* = 0.044, *d* = 0.55. Again, this effect persists when Years in Education is included as a covariate – *F*(1,55) = 6.706, MSE = 0.191, *p* = 0.012, with Education having a weak but non-significant effect (*p* = 0.099)^1^.

For explicit questions regarding immigration there was a weak relationship with IAT scores. Higher IAT bias scores were associated with feeling that immigration levels were too high [Pearson’s *r*(160) = 0.192, *p* = 0.015]. However, lower IAT scores were not strongly associated with the belief that wealthier nations should take more refugees [*r*(160) = 0.112, *p* = 0.16]. Finally greater support for the death penalty was weakly associated with higher IAT scores – *r*(160) = 0.169, *p* = 0.032.

### Exploratory Results

We first considered participants voting behavior in the EU Referendum, where voting preferences have been frequently linked with attitudes to immigration. Daily Mail (59%) readers were more likely to say they had voted to leave the EU than Guardian readers (15%) – *F*(1,158) = 28.461, MSE = 0.321, *p* < 0.001, *d* = 0.85. However, performing an ANCOVA with Newspaper as a fixed factor and treating Brexit Leave/Remain vote as a covariate revealed a significant effect of choice of newspaper – *F*(1,157) = 3.94, MSE = 0.149, *p* = 0.049, but no reliable effect of leave/remain vote as a covariate – *F*(1,157) = 0.437, MSE = 0.149, *p* = 0.509. Nonetheless, leave voters (*n* = 58) did tend to have higher overall IAT scores (*M* = 0.89, *SD* = 0.37) compared to remain voters (*n* = 88 – *M* = 0.79, *SD* = 0.40), although this difference was not reliable – *F*(1,144) = 2.296, MSE = 0.152, *p* = 0.132). Fourteen participants indicated they did not vote in the Brexit referendum, with those participants showing slightly higher bias scores (*M* = 1.01, *SD* = 0.32) than remain (*t*(100) = 1.989, *p* = 0.061) and leave voters: *t*(70) = 1.068, *p* = 0.289.

We also explored if broader political party preference was associated with implicit bias scores. We entered Newspaper as a predictor into a linear regression (step 1), followed by dummy-coded political party preference (step 2). No political party preference was retained as part of the final model, indicating that political party preference was not a reliable predictor of implicit bias scores. In a similar vein, adding Gender as a predictor to a model that already contained Newspaper did not improve fit. However, adding Age as a predictor to a model also containing Newspaper did provide for better overall model fit – *R*^2^ = 0.06, *F*(2, 157) = 5.02, *p* = 0.008, with older ages associated with an increase in bias scores *b* = 0.005, *t*(157) = 2.528, *p* = 0.012.

Choice of newspaper was also associated with people’s explicit attitudes to immigration, although not consistently. Daily Mail readers (*M* = 4.13, *SD* = 0.789) rated immigration levels as too high compared to Guardian readers (*M* = 3.310, *SD* = 0.791) – *F*(1,158) = 43.191, MSE = 0.624, *p* < 0.001, *d* = 1.04. Daily Mail readers (*M* = 2.224, *SD* = 1.218) rated similarity to immigrants as lower than Guardian readers (*M* = 3.048, *SD* = 1.097) – *F*(1,158) = 20.282, MSE = 1.335, *p* < 0.001, *d* = 0.72. However, there was no reliable difference in how Daily Mail readers (*M* = 1.263, *SD* = 0.443) and Guardian readers (*M* = 1.179, *SD* = 0.385) felt about wealthier nations taking more refugees – *F*(1, 158) = 1.667, MSE = 0.171, *p* = 0.199, *d* = 0.2.

## General Discussion

In a pre-registered study we found that readers of the Daily Mail newspaper exhibited a larger IAT bias, revealing stronger negative associations to immigration concepts compared to readers of The Guardian newspaper. This difference in implicit associations was consistent with linguistic distributional differences for immigration terms on those newspapers’ websites, where the IAT immigration stimuli had a stronger negative association for the Daily Mail compared to the Guardian, and stronger positive association for the Guardian compared to the Daily Mail. The difference in IAT scores was not explained by readers’ level of education, or by other variables in exploratory analyses (e.g., EU referendum vote, political party preference, gender).

While the overall size of the effect was small-to-moderate (*d* = 0.31), as Greenwald et al. (2015) note, even smaller effects in the IAT can reflect large effects at the societal level. Furthermore, the effect size increases to *d* = 0.5 or higher when we consider only high-frequency readers of the newspapers. Importantly, in our exploratory analyses, the difference in overall bias was not reliably explained by political party preferences, vote in the Brexit referendum, or gender. However, the study was not designed to fully test for these various covariates and their interactions, and it may be that these and other factors still have a role to play, which will be more fully examined in future work.

### Limitations

While the findings are consistent with the view that exposure to biased language may lead to biased attitudes, it is important to note that we are merely showing an association between linguistic regularities and implicit attitudes, and not demonstrating a causal relationship between exposure to biased language and the subsequent development of biased attitudes. Nonetheless, we learn through associations encountered via what we read, hear, and see, and it may be that reading newspapers can reinforce or enhance our pre-existing biases, or indeed plant the seeds for new biases to flourish. It is possible, and indeed plausible, that people’s pre-existing biases may lead them to consume particular news sources - in other words, those with stronger anti-immigration implicit biases are more likely to choose to read the Daily Mail over the Guardian. If this is the cases, it remains possible that the further consumption of a chosen media sources could form part of a causal cycle. That is, people initially select newspapers based on pre-existing biases (see e.g., Galdi et al., 2012), but that those biases are then compounded by the subsequent increased exposure to biased language. Importantly, demonstrating the association between linguistic patterns and implicit attitudes is an important precursor to identifying the causal nature of the relationship. To extend this work, future studies could attempt to disentangle the precise relationship by systematically varying the news content that people are exposed to, and looking at the impact specifically on implicit attitudes. Such studies could also be supplemented with additional measures to more fully investigate the effects of political ideology, and perhaps differences between conservatives and liberals in how they respond to information that is consistent/inconsistent with their beliefs (see e.g., Gampa et al., 2019).

However, there are limitations of the current study that should also be noted when considering the results, notably issues of statistical power, and the need for further investigation of exploratory findings. Following exclusions, the overall sample size was reduced from 209 to 160 participants, which meant our overall statistical power was also reduced. Given that the observed overall effect size was approximately *d* = 0.3, future studies would need to have a sample size of at least 352 participants (following exclusions) to have 80% statistical power (G^∗^Power: Faul et al., 2009) of detecting such an effect. Furthermore, if we wish to follow-up on our exploratory analysis (e.g., links between bias and voting patterns or political preferences), sample sizes would need to be further increased to incorporate covariates or other more complex statistical models.

Of course, the newspapers people read reflect only a fraction of the information people consume, and there are many other factors operating at various levels (i.e., individual, group, societal), that can influence people’s implicit biases. As well as differences in individual experience (e.g., media consumption, family, and education), membership of different groups, and culture-specific representations of others, can each contribute to the formation and development of implicit biases. In terms of individual experience, Ranganath and Nosek, 2008 found that even a short period of training about negative behaviors performed by individuals lead to negative implicit associations about those individuals, but also that those biases were generalized to other members of the same group (see also Kawakami et al., 2000; Ashburn-Nardo et al., 2001; Paladino and Castelli, 2008). In terms of group membership, it has long been known that people’s attitudes and behaviors toward others vary according to whether they are considering in-group or out-group members (e.g., Brewer, 1979; Tajfel et al., 1979). Such differences in attitudes also extend to implicit biases. For example, in the United States, there is ample evidence that implicit biases from white physicians is related to substandard treatment of non-white patients relative to white patients (Green et al., 2007; Penner et al., 2010), while Black physicians tend not to show any difference in implicit bias toward White or Black Americans.

Broader cultural experience is also going to impact the development and activation of implicit biases. Examining Native Hawaiians’ implicit associations between race and place, Godsil and Freeman (2015) note that “cultural ideology” (e.g., Native Hawaiian culture places great importance on the relationship between people and the land) can complicate the operation of implicit biases. We can also explicitly link language and cultural experience – Danziger and Ward (2010) found that when Arab-Israeli participants completed an Arab-Jew IAT, their pattern of responses differed depending on whether they complete the task in Hebrew or Arabic. That is, weaker pro-Arab associations were observed when the task was completed in Hebrew compared to when it was completed in Arabic. Thus, beyond the linguistic input people consume, which we examine in this paper, a complete account of implicit biases will necessarily need to take a much broader perspective.

## Conclusion

These findings raise the possibility that exposure to language that is biased for and/or against immigration contributes to implicit attitudes toward immigration. We already know that media exposure affects other attitudes, such as increased advertising exposure linked to greater product liking and purchases (Vakratsas and Ambler, 1999). More broadly, the findings suggest that, in accounting for biased decision making, one must entertain the possibility that people may rely on statistical regularities from the linguistic environment, rather than on any deeper cognitive processing. In line with work by Caliskan et al. (2017), the findings also reinforce the need for caution when developing computational decision-making systems trained on natural language corpora, which can be imbued with cultural biases present in the statistical regularities of the corpora and therefore “behave” in a prejudicial manner as a consequence.

The findings also raise potential ethical issues for journalists and the media – if the goal of the media is to report news, then biased language should be avoided. However, if the purpose of the media is to influence consumer attitudes, then perhaps consumers need to be more aware of the possible effects the media’s linguistic choices might have on them.

## Ethics Statement

This study was carried out in accordance with the recommendations of Lancaster University’s Faculty of Science and Technology Research Ethics Committee. All subjects gave informed consent in accordance with the Declaration of Helsinki. The protocol was approved by the Lancaster University Faculty of Science and Technology Research Ethics Committee.

## Author Contributions

DL, MW, LC, LCr, KOB, and TM designed the study and wrote the manuscript. LCr and DL created the materials, developed the experiments, collected and analyzed the data.

## Conflict of Interest Statement

The authors declare that the research was conducted in the absence of any commercial or financial relationships that could be construed as a potential conflict of interest.

## Footnotes

1 The analyses for frequent readers where Years in Education is treated as a covariate were not part of our pre-registered analysis plan, but for clarity we include them adjacent to the non-covariate versions of the analyses
