# Supplementary material for: Are You What You Read? Predicting Implicit Attitudes to Immigration Based on Linguistic Distributional Cues From Newspaper Readership; A Pre-registered Study
Source: Front Psychol. 2019 May 3;10:842. doi: 10.3389/fpsyg.2019.00842 (PMC6509147; doi:10.3389/fpsyg.2019.00842)
Supplement: Supplementary file 1 [file Data_Sheet_1.PDF]

Supplemental Information

This document contains:

Additional materials used in the immigration IAT study, namely Explicit Questions and Demographic Information

1. Explicit Questions and Demographic Information

To what extent do you think UK immigration levels are too high? (far too low; too low; just the right level; too high; far too high)

Do you agree that those countries with greater resources should allow asylum to more refugees (yes; no)

To what extent do you feel similar to immigrants to your county? (very dissimilar; slightly dissimilar; neither dissimilar nor similar; slightly similar; very similar)

Did you vote to leave or remain in the recent (2016) EU membership referendum? (leave; remain; did not vote)

Which political party did you vote in the last general election? (Labour Party; Conservative Party Liberal Democrats; Green Party; UK Independence Party; Other Party)

How much do you agree or disagree with the following statement: "For some crimes, the death penalty is the most appropriate sentence" (Strongly disagree; Disagree; Neither agree nor disagree; Agree; Strongly agree|)

Which of the following newspapers do you read and how often? (The Daily Express; The Daily Mail; The Financial Times; The Guardian; The Independent; The Sun; The Telegraph; The Times – daily; more than once per week; once per week; occasionally; never)

How much would you say you trust the following newspapers for their news content? (The Daily Express; The Daily Mail; The Financial Times; The Guardian; The Independent; The Sun; The Telegraph; The Times - trust completely; trust somewhat; neither trust nor distrust; distrust somewhat; distrust completely)

Please list any other news sources you regularly use

What is your gender? (male; female; other; prefer not to say)

What is your age?

Were you born in the UK? (Yes; No)

What is the highest level of education you have completed? (no formal schooling; primary school; secondary/high school; further education; Bachelor's degree; Master's degree; Doctoral degree)
